# Studies of Infection and Experimental Reactivation by Recombinant VZV with Mutations in Virally-Encoded Small Non-Coding RNA

**DOI:** 10.3390/v14051015

**Published:** 2022-05-10

**Authors:** Punam Bisht, Biswajit Das, Tatiana Borodianskiy-Shteinberg, Paul R. Kinchington, Ronald S. Goldstein

**Affiliations:** 1 Mina and Everard Goodman Faculty of Life Sciences, Bar-Ilan University, Ramat-Gan 52900, Israel; punam.bisht.pb@gmail.com (P.B.); biswamicrobio@gmail.com (B.D.); shteinbergt@gmail.com (T.B.-S.); 2 Departments of Ophthalmology and of Microbiology and Molecular Genetics, University of Pittsburgh, Pittsburgh, PA 15213-2588, USA; kinchingtonp@upmc.edu

**Keywords:** varicella zoster virus, latency, reactivation, human neuron culture, non-coding RNA

## Abstract

Locked-nucleotide analog antagonists (LNAA) to four varicella zoster virus small non-coding RNA (VZVsncRNA 10–13) derived from the mRNA of the open reading frame (ORF) 61 gene individually reduce VZV replication in epithelial cells and fibroblasts. To study the potential roles VZVsncRNA 10–13 have in neuronal infection we generated two recombinant VZV; one in which 8 nucleotides were changed in VZVsncRNA10 without altering the encoded residues of ORF61 (VZVsnc10MUT) and a second containing a 12-nucleotide deletion of the sequence common to VZVsncRNA12 and 13, located in the ORF61 mRNA leader sequence (VZVsnc12-13DEL). Both were developed from a VZV BAC with a green fluorescent protein (GFP) reporter fused to the N terminal of the capsid protein encoded by ORF23. The growth of both mutant VZV in epithelial cells and fibroblasts was similar to that of the parental recombinant virus. Both mutants established productive infections and experimental latency in neurons derived from human embryonic stem cells (hESC). However, neurons that were latently infected with both VZV mutant viruses showed impaired ability to reactivate when given stimuli that successfully reactivated the parental virus. These results suggest that these VZVsncRNA may have a role in VZV latency maintenance and/or reactivation. The extension of these studies and confirmation of such roles could potentially inform the development of a non-reactivating, live VZV vaccine.

## 1. Introduction

Human Herpesvirus-3 or Varicella-Zoster virus (VZV) is a neurotropic alphaherpesvirus that causes varicella (chickenpox) after primary infection, and then establishes a life-long latent infection of peripheral ganglionic neurons [1]. Reactivation from latency in response to poorly defined stimuli and/or immune decline results in the development of reactivation disease, usually manifesting as painful Herpes Zoster (shingles; HZ). HZ is frequently complicated, most often by chronic pain states extending beyond the resolution of the infection termed postherpetic neuralgia or PHN [2]. Most non-human species and cells derived from them do not support full productive VZV infection, except the guinea pig whose cells were used to attenuate a clinical virus (Parent of Oka or POka) and generate the vOka vaccine strain [3]. The human-specificity of VZV infection and consequent lack of animal models has hampered the study of VZV latency and reactivation. This is in contrast to the ready in-vitro and in-vivo experimental infection of rodent and rabbit neurons by the related alphaherpesvirus herpes simplex that has allowed myriad studies of these processes for HSV1/2. 

However, three recent experimental developments have permitted the advancement of our understanding of these clinically important processes. The first was the development of models of human neuronal infection, such as human ganglionic tissues in culture or grafted to severe compromised immunodeficient mice (SCID-hu DRG mice (reviewed in [4]). The second was the development of experimental models using human stem cell-derived neurons that recapitulate VZV latent infection in-vitro that can be experimentally reactivated [5]. The use of these models has revealed, for instance, that the vaccine strain vOka, infects neurons latently but is impaired for reactivation [6]. They have also been used to demonstrate that the Jun N- terminal kinase (JNK) pathway contributes to the lytic/latent decision process [7]. The third important advance was the discovery of a family of novel transcripts termed VLT in human ganglia obtained from VZV seropositive individuals at death. These are positionally and directionally similar to the herpes simplex virus (HSV) latency-associated transcript (LAT) and latency transcripts reported for other neurotropic alphaherpesviruses [8]. Multiple splice variants of VLT are found in cultured cells productively infected with VZV and evidence has been presented that unlike LAT of HSV, some spliced variants of VLT can encode proteins. It is still not clear how VZV VLT and the long-studied HSV LAT contribute to the infection of neurons, the latent state, and reactivation from it. 

A focus of recent research has been the potential role of virally and host-encoded small non-coding RNAs (sncRNA), particularly microRNA (miR). Of the nine human herpesviruses, seven have been reported to express miRNA, with at least 29 miRNAs being reported to be encoded by HSV-1 [9]. The roles of several HSV-1 miRNAs have been evaluated for their potential contribution to productive and latent infection of neurons and the lytic/latent decision process (i.e., [10], reviewed in [11]). In addition to miRNA, other small RNAs such as sncRNA have been reported to be expressed in mammalian cells that can also regulate gene expression at the transcriptional level [12]. 

The encoding of many miR by the human herpesviruses, and especially the alphaherpesviruses HSV1 and HSV2, led us to search for sncRNA in VZV. We first predicted that 24 20–24 nt small non-coding sncRNA (VZVsncRNA to be encoded by the VZV genome [13] from bioinformatic analysis of NGS data of small (>200 nt) RNA in VZV-infected cells. Stem-loop reverse-transcriptase quantitative TaqMan PCR (SL-rt-qPCR) detected 23 of 24 of these sncRNA in multiple infected cell types in culture [14]. Transfection of locked nucleotide analog antagonists (LNAA) to several of the VZV-encoded small RNA sequences (VZVsncRNA) significantly reduced viral spread, suggesting that the predicted VZVsncRNA may have roles in VZV infection. 

More recently, we found that LNAA directed to VZVsncRNA10–13, 4 VZVsncRNA that are encoded by the mRNA for the ORF61 gene and opposite to the putative VZV latency-associated transcript VLT, consistently reduced both the viral spread and plaque number [15]. LNAA-mediated inhibition of VZV sncRNA12 not only decreased viral replication but also resulted in a significant increase in VLT levels expressed during lytic infection. ORF61 encodes a protein that is the VZV homolog of HSV ICP0, a pro-lytic non-specific transactivator that functions in part to counteract intrinsic and innate responses to HSV infection through an E3 ubiquitin ligase activity [16]. Much stronger anti-viral effects were observed when combinations of antagonists to these four VZVsncRNA were found in the epithelial cell-line ARPE-19 and in primary human fibroblasts [17]. 

In view of the proposed roles of miR encoded by LAT in HSV1 reactivation, we here experimentally address the possibility that VZVsncRNA10–13 may influence VZV latency and reactivation. Two VZV mutant BACs were generated in which the sequences encoding for VZVsncRNA10 and VZVsncRNA12/13 were altered without changing the coding of ORF61 using BAC recombineering methods. The two VZV mutant viruses derived appeared not to result in significant differences in productive infection of either ARPE19 cells or in cultured neurons when compared to their parent VZV. Both mutant viruses also established experimental latency in hESC-derived neurons including the silencing of lytic gene expression, the expression of VLT RNAs, and the presence of viral genomes detected by PCR. When neurons latently infected by both VZVsncRNA mutant viruses were challenged with stimuli that experimentally reactivate VZV, their reactivation appeared impaired compared to cultures of the neurons latently infected with the parental VZV. These data are consistent with the possibility that these VZVsncRNA, which are encoded opposite to VLT, may have a role in the VZV latency/reactivation switch.

## 2. Materials and Methods

### 2.1. Cells 

MeWo (human melanoma cell line ATCC HTB-65) were cultured in DMEM, containing 10% FBS, 2 mM glutamine, 100 U/mL penicillin, 100 µg/mL streptomycin, and 0.25 µg/mL amphotericin B. ARPE19 (human retinal pigmented epithelium (ATCC CL4000) were maintained in DMEM F12 with the glutamine and antibiotics. 

### 2.2. Neuronal Differentiation from hESC

The human embryonic stem cell (hESC) line H9 (WA09 (WiCell Madison WI, USA)) was maintained on murine embryo fibroblast line (ATCC CRL-1503) in Nutristem (Biological Industries, Israel) medium and differentiated into neurons using a modification of the agarose microwell method of Birenboim et al. [18]. Briefly, hESC were dissociated with Accutase (Sigma-Aldrich, St. Louis, MO, USA), and 1 × 10^6^ cells were seeded into 256-well agarose microwell dishes made from silicone molds (Sigma-Aldrich). The cells aggregated for 4 days in the molds in a medium consisting of GMEM (Gibco/Life Technologies, Carlsbad, CA, USA) 1% penicillin/streptomycin (Biological Industries (BI), 1% L-glutamate (BI) 1% pyruvate (BI) 10% KSR—knockout serum replacement (Gibco) 1% non-essential amino acids (BI) 0.1 um mercaptoethanol (Sigma-Aldrich), containing bone morphogenetic protein (BMP)4 inhibitors SB431542 (10 μM) and dorsomorphin (2 μM) (Tocris Bioscience). The aggregates were then fed for an additional 10 days in a medium lacking the BMP4 inhibitors. The aggregates were then plated on coverslips or wells of 24 well plates coated with polylysine/laminin or tissue culture plates in a differentiation medium consisting of DMEM/F12 with neural growth and survival factors NGF, BDNF, NT3, and GDNF (Alomone Labs) and B27 supplement (Gibco). Approximately 10–20 aggregates were plated on each coverslip/well. Dividing cells were eliminated from the cultures 2 days after seeding using mitotic inhibitors 24 µM 5-fluorodeoxyuridine (F0503, Sigma-Aldrich) and 0.6 µM cytosine arabinoside (C6645, Sigma-Aldrich) added 4 days after plating. Twenty-four µM Uridine (U3750, Sigma-Aldrich) was added to offset the toxicity of the fluordeoxyuridine ([19,20]). Cultures were maintained for a total of 21 days after removal from the molds. Immunofluorescent staining for neurofilament proteins was performed with antibody 2H3 (NF-M) deposited to the Developmental Studies Hybridoma Bank by Jessell, T.M. and Dodd, J., and Sigma-Aldrich (Merck) N4124 (NF-H).

### 2.3. Generation of VZV Mutant in VZVsncRNA

The two VZV mutant viruses were generated using a bacterial artificial chromosome (VZV BAC) based on the parent Oka (POka strain [21], that contained EGFP fused to the N terminus of ORF23 (VZVORF23GFP) detailed previously [12] and housed in the *E. coli* strain GS1783 (kind gift of G Smith Northwestern University, Chicago, IL, USA). GS1783 contains heat-inducible expression of the λ red recombination genes and an arabinose inducible expression of the homing enzyme IsceI. Briefly, primers developed to contain the sncRNA10 mutant or sncRNA 12/13 deletion were used to amplify a kanamycin resistance cassette from the plasmid pEGFP KAN-in [13] using the primers listed in Table 1.

The amplification products were PCR amplified and then recombined into VZV BAC GFP23 after electroporation as detailed previously [21] and modified by Erazo et al. [22]. Colonies selected on LB-agar plates containing kanamycin + chloramphenicol were then screened for correct insertion by RFLP analyses and then subjected to a second recombination event concurrent with arabinose induction of the expression of ISceI to counterselect for loss of the kanamycin resistance cassette. Clones were again screened for removal of the cassette by RFLP analyses and sequencing across the junctions. Selected colonies were stored as 50% glycerol stocks. 

Mutant VZV (VZVsnc10MUT and VZVsnc12-13DEL) were derived from the BACs following transfection of BAC DNA into MeWo cells using Lipofectamine 3000 (Thermo-Fischer Scientific, Waltham, MA, USA), as detailed in [22]. The viruses were verified for homogeneity of green fluorescence, which was found to be stable over extensive passaging so that all plaques remained GFP positive. Cell-free and debris VZV for infection were generated as detailed previously [5].

### 2.4. Stem-Loop Taqman qPCR for VZVsncRNA Quantification and Detection

The expression of the VZVsncRNA was carried out by Stem-loop TaqMan qPCR as detailed previously [13]). In brief, small (<200 nt) RNA obtained from ARPE19 cells infected with cell-free VZV were extracted using the Hybrid-R kit (Geneall, Seoul, Korea). Primers were designed using published algorithms [23] and the probe used was LGC Biosearch Technologies # DLO-RFB-5 (Middlesex, UK)) (Table 2). cDNA for VZVsncRNA10mut or VZVsncRNA12-13 as well as hsa-mir26 were prepared in separate reactions using MMLV reverse transcriptase (Promega, # M1701). RNA was treated with DNase (AMPD1, Sigma-Aldrich). All qPCR reactions were performed in triplicate and results were averaged to compensate for pipetting errors. Human hsa-mir26 shows little variation in expression between cells infected and uninfected by multiple viruses [4] and was used to normalize the expression of small RNA.

### 2.5. qRT-PCR for Viral Transcripts

Total RNA was extracted using the Hybrid-R kit for large RNA/small RNA (Geneall, Seoul, Korea) from hESC-derived neurons that were mock-infected or infected with 100 PFU of cell-free VZV23GFP, VZVsnc10MUT or VZVsnc12-13DEL Infections were monitored using GFP expression indicating productive infection for 7 days, or from neurons infected with 100 PFU of cell-free VZV23GFP, VZVsnc10MUT or VZVsnc12-13DEL ACV for 10 days and without ACV for 15 days that did not express GFP (latently infected). RNA was treated with DNase (AMPD1, Merck, Darmstadt, Germany). For the cDNA synthesis, 3 μg of total RNA was reverse transcribed using an oligo dT primer and Moloney murine leukaemia (M-MMLV) reverse transcriptase (# M1701; Promega, Madison, WI, USA). Negative control samples were obtained by performing the same cDNA synthesis reaction in the absence of reverse transcriptase. Three independent biological experiments were performed, and qPCR was performed in triplicates for each target. Primer- probe- probe sets for the VZV genes ORF 61, ORF 63, ORF 31, and VLT3F-4R sequence are listed in Table 1. Results are shown as averages with error bars representing the SEM.

### 2.6. Latency/Reactivation Experiments

After a minimum of 21 days of terminal differentiation of neurons into cultures that contained extensive axonal outgrowth, cells were pre-treated with acyclovir (ACV, 400 uM) for 24 Hrs, and then incubated with cell-free VZV (VZVsncRNA10 or VZVsncRNA12-13DEL and VZV23GFP). Cultures were exposed to 100 PFU of the virus in the presence of 400 uM of ACV and centrifuged at 180 g for 10 min, followed by incubating for a period of 2 Hr. at 37 °C. After the removal of the virus, the neurons were maintained in the presence of ACV for 10 days with daily media changes. Media without the ACV was then used to maintain the cultures for up to 2 weeks post-infection, with media changes every 3 days. Cultures were examined regularly over the incubation period for GFP expression microscopically, but no GFP fluorescence was observed. After two weeks, latently infected wells were subjected to a reactivation stimulus involving treating the neurons with LY294002 hydrochloride (10 μM, Tocris, CAT. #1130) and phorbol ester phorbol 12-myristate 13-acetate (PMA) (40 ng/mL) and were incubated at 33 °C for 5d. Reactivation of virus in live cultures was detected as GFP fluorescence representing the expression of ORF23. Three biological repeats of latency experiments each with 3 latently infected wells were carried out for each mutant with parallel VZVGFP23 controls. To confirm that GFP expression corresponded to reactivation including production of infective virions, 7d after application of the chemical stimuli the neurons were harvested and seeded on naïve ARPE19 cells, and infectious foci were observed using GFP fluorescence. 

### 2.7. Multistep Growth-Kinetics

The multistep growth kinetics of VZVsncRNA10 or VZVsncRNA12-13DEL and VZV23GFP viruses were determined by inoculating ARPE19 cells with 1000 PFU of VZV debris-fraction. Infected cells were subsequently trypsinized at 2 h (“time 0”) or 1, 2, 3, 4, and 6 dpi, serially diluted, and titrated by seeding 10-fold dilutions of harvested cells onto fresh ARPE19 monolayers. Seven days after seeding, viral plaques were fixed with 4% (*w*/*v*) paraformaldehyde containing crystal violet for 30 min and plaques were counted.

## 3. Results

### 3.1. Construction of VZV with Synonymous Mutation of VZVsncRNA10 and Deletion in the Overlap Region between VZVsncRNA 12&13

**Figure 1:**
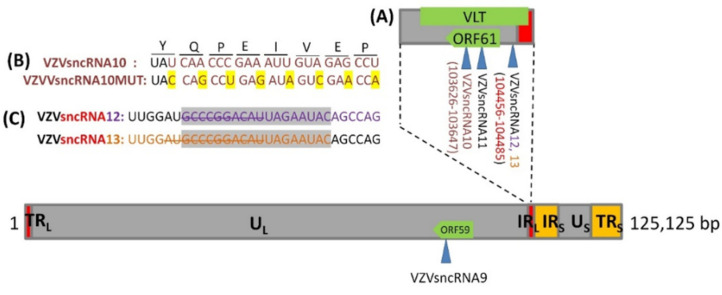
Genomic localization of VZVsncRNA10, VZVsncRNA12 VZVsncRNA13 expression and mutagenesis (**A**) depicts the region of the VZV genome containing the VZVsncRNA studied. Arrowheads indicate the VZVsncRNA coded by the lower viral DNA strand and deriving from a right to left primary transcript that is predicted to encode ORF61. (**B**) Mutations introduced into VZVsnc10MUT: the upper sequence shows the original sequence while the lower sequence shows the synonymous mutations introduced by mutagenesis highlighted in yellow. (**C**) Sequences of VZVsncRNA12 (purple letters) and VZVsncRNA13 (orange letters) deleted in VZVsnc12-13DEL l are indicated with a strikeout. The portion of VZVsncRNA12 and VZVsncRNA13 that overlap is highlighted in gray. The sncRNA is shown in the correct direction.

Transfection of LNAA antagonists to VZVsncRNA10, 12, and 13, singly or in combinations, significantly reduces VZV productive infection. This suggests that the VZVsncRNA themselves modulate lytic infection by VZV [15]. In order to further study the roles of these VZVsncRNA in VZV infections, two recombinant VZV mutants’ viruses were derived in the background of a VZV BAC that contains a GFP reporter gene fused to ORF23, previously shown capable of establishing lytic infections in hESC-derived human neurons and model VZV latency and experimental reactivation ([6,24]). The BACs were generated by λred-mediated recombineering methods used in our laboratories [25]. The VZVsncRNA10 was mutated by changing 8 nt without altering the ORF61-encoded amino acid sequence, yielding a virus termed VZVsnc10MUT. The second recombinant VZV contained a deletion of 12 of the nucleotides common to VZVsncRNA12 and 13 that are located immediately upstream of the ORF61 coding sequence and presumably lies in the ORF61 mRNA non-coding leader. VZV derived from this BAC was termed VZVsnc12-13DEL (Figure 1A,B). All mutated sequences were confirmed in the BACs by sequencing across the junctions. 

**Figure 2:**
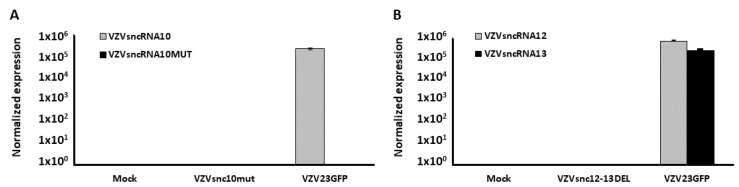
VZVsncRNA10MUT and VZVsncRNA12-13DEL do not express the corresponding sncRNA in VZV-infected cells. ARPE19 cells were infected with a cell-free virus from VZV23GFP (parental virus), VZVsnc12-13DEL, or VZVsncRNA10MUT. RNA size-selected for <200 nt was extracted at 5 dpi and VZVsncRNA10, VZVsncRNA10MUT, VZVsncRNA12, and VZVsncRNA13 expression was assayed by SL-qRT-PCR. (**A**) shows the expression of VZVsncRNA10 for infections with VZVsncRNA10mut and parental virus and (**B**) shows the expression data for VZVsncRNA12 and VZVsncRNA13 in cultures infected with VZVsncRNA12–13DEL and parent viruses. Expression is normalized to cellular miR26, whose expression has been shown not to be affected by viral infection [4].

Both mutation strategies resulted in BACs that yielded viable VZV. Initial characterization of the mutant VZV was performed by infecting ARPE19 cells with each virus, extracting RNA at 5 dpi, and subjecting it to Taqman stem-loop quantitative reverse transcriptase PCR (SL-qRT-PCR) for the sncRNA, using primers matching the native and mutated sequences [13]. As expected, VZV sncRNAmut10 infected cells did not show any expression of VZVsncRNA10, while cells infected with VZV derived from the parental BAC showed strong expression (Figure 2A). In addition, stem-loop primers directed to the mutated sequence did not result in PCR amplification from RNA of cells infected with the mutant virus (or the parent virus) suggesting that either the mutated sequence was not expressed as an alternative sncRNA or that it was rapidly degraded in the host cells. Similar assays of RNA from cells infected with VZVsnc12-13DEL indicated complete abrogation of expression of both VZVsncRNA12 and VZVsncRNA 13 compared to parent control virus when sncRNA12 and 13 specific primers were used (Figure 2B), and these also detected abundant levels in another pOka-derived virus, VZV66GFP (Figure 2 and Refs. [13,14]). 

### 3.2. Growth Rates of VZVsnc10MUT and VZVsnc12-13DEL Are Similar to Those of Their Parental Virus in ARPE-19 Cells

**Figure 3:**
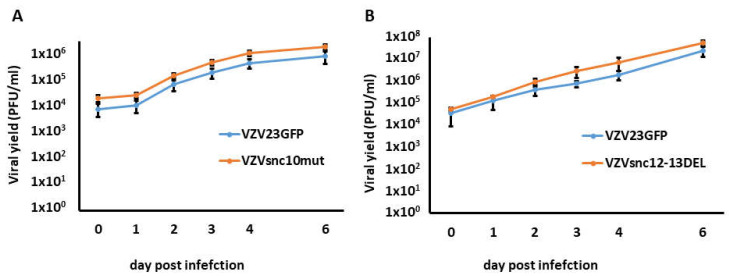
VZVsnc10MUT or VZVsnc12-13DEL growth curves compared to VZVGFP23 in lytically infected ARPE-19 cells. ARPE19 cells were infected with VZVsncRNA10mut or VZVsnc12-13DEL debris [5] and subsequently harvested from one well by trypsinization. Cells were then serially diluted and titrated for time 0 (0 h) or subsequent times into fresh monolayers of ARPE-19 cells. At 7 dpi, the cells were fixed and stained for 30 min, and the plaques were counted on dried and stained plates to give values of viral yield in PFU. (**A**) Represents the growth of VZV23GFP compared to VZVsncRNA10mut and (**B**) represents the growth of VZV23GFP compared to VZVsnc12-13DEL. *n* = 3 independent experiments for each mutant virus.

As mentioned above, antagonizing VZVsncRNA10,12 and 13 with LNAA reduced viral spread and generation of infectious viruses [15]. This raised the possibility that mutating their sequences could affect viral replication. We therefore conducted growth curve analyses for the mutants after a cell-free infection with a cell-free viral “debris” fraction (cellular debris derived from VZV-infected cells containing high-titers of infectious virus but no living cells [5]). The growth curves were generated from standard plaque assays and compared to those obtained from the parental VZVGFP23 virus. Both VZVsnc10MUT (Figure 3A) and VZVsnc12-13DEL (Figure 3B) displayed similar replication kinetics over a 6d period yielding a slight but not significant increase in infectious virus production in comparison to those of the parental VZV23GFP. 

### 3.3. VZVsnc10MUT and VZVsnc12-13DEL Can Productively Infect Human Embryonic Stem Cell-Derived Neurons

**Figure 4:**
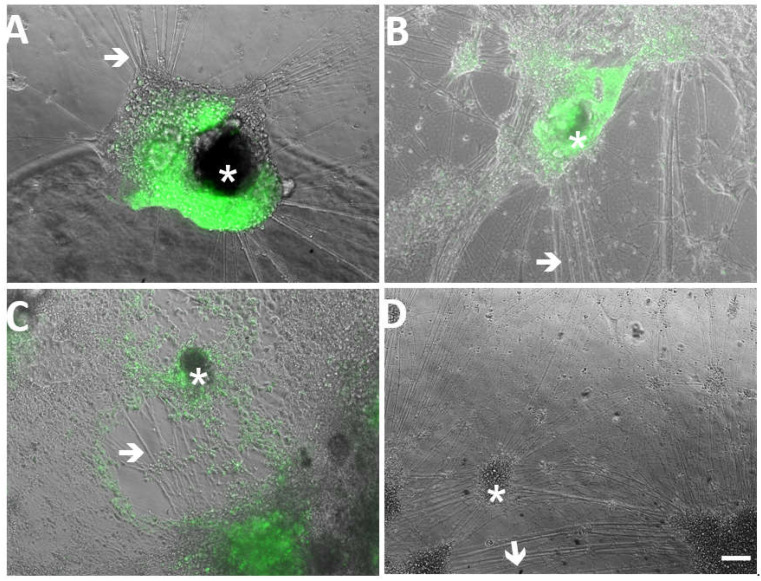
hESC-derived neurons are productively infected by recombinant VZV lacking expression of VZVsncRNA10 and VZVsncRNA12 and 13. Panel A-D show merged images of GFP fluorescence and phase contrast. Neurons differentiated for 21d were infected with cell-free VZV23GFP, VZVsnc10MUT or VZVsnc12-13DEL virus. Extensive productive infection of neurons was observed at 5 dpi for VZVsnc10MUT (**A**), VZVsnc12-13DEL (**B**) and VZV23GFP (**C**) as shown by expression of ORF23GFP. (**D**) Shows neurons in a mock-infected well. Arrowheads indicate neurites and asterisks indicate clusters of neuronal cell bodies. Scale Bar = 100 mm.

We next addressed the question of whether neurons, the reservoir of latent VZV after varicella, supported productive infection by recombinant viruses with mutations in ORF61-derived VZVsncRNA. Human embryonic stem cell (hESC)-derived neurons were made using agarose micromolds (as described previously [18]) and then matured for 1 month on coverslips in 24-well plates. Neuronal cultures were then exposed to 100 PFU of cell-free VZVsnc10MUT, VZVsnc12-13DEL or VZV23GFP and neuronal infection was monitored by observing GFP fluorescence microscopically indicating the synthesis of VZVORF23 capsid protein. An extensive, spreading productive infection was observed for all three viruses, shown at 7 dpi in Figure 4A–C. As expected, no fluorescence was observed in mock-infected neurons (Figure 4D). This result indicates that the mutation/deletion of these VZVsncRNA does not prevent infection leading to viral replication of VZV in human neurons. Unfortunately, the heterogenous nature of our hESC-derived neuron cultures in terms of both number and neuronal phenotypes precludes performing growth curve analyses for the mutant viruses in neurons as we did in ARPE19 cells and fibroblasts.

### 3.4. VZV Mutated for VZVsncRNA Derived from the mRNA for ORF61 Can Establish Experimental Latency in hESC-Derived Neurons

VZVsncRNA10-12 are derived from the mRNA for ORF61 and would be complementary to the recently identified VLT transcript expressed in cadaveric dorsal root ganglia of VZV- seropositive individuals. VLT has positional and antisense activities similar to other herpesvirus latency-associated transcripts and has been implicated to be involved in neuronal latency and/or reactivation [26]. In order to determine if a latency-like infection could be established by the mutant VZV in neurons, we used a model previously used by us [6] and others [27] in which acyclovir (ACV) treatment is used to inhibit lytic replication after infection of neuronal cultures with a cell-free virus. 

**Figure 5:**
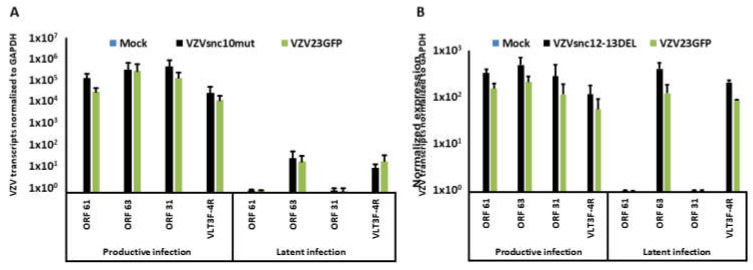
VZV mutant in VZVsncRNAs establishes experimental latency in hESC-derived neurons. (**A**) hESC-derived neurons were infected with 100 PFU of cell-free VZV. Latent infections were obtained by treatment with ACV (see methods for exact protocol). In the absence of ACV, all three viruses infected neurons productively (see Figure 4). RNA was harvested at 5 dpi from the wells not treated with ACV and at 25 dpi from presumably latently infected neurons in wells treated with ACV and lacking GFP expression. RT-qPCR analysis for IE transcripts ORF61 and ORF63, late transcript ORF31, and transcripts of VLT exons 3 and 4 of VLT VLTexon3–4 (4) was then performed. (**A**) VZVsnc10mut productively infected neurons (left 4 sets of bars) when not treated with ACV. ACV-treated latently infected wells (right 4 sets of bars) expressed very low levels of ORF61 and late transcript ORF31 consistent with our previous report [6]. The only transcripts detected at higher levels were those of VLT exons 3 and 4 of VLT (VLTexon3–4) (4) and a portion of ORF63 that is co-expressed with VLT [7] consistent with the establishment of latency. *N* = 3 independent experiments. (**B**) RT-qPCR analysis of ACV-untreated, productively infected (left 4 sets of bars) and ACV-treated, latently infected (right 4 sets of bars) neurons infected with VZVsnc12-13DEL revealed the same expression patterns of transcripts as observed for infections with VZVsnc10mut. That is, neurons productive infected with VZVsnc12-13DEL expressed all 4 tested transcripts at high levels, while the latently infected neurons only expressed transcripts expected to be present in latency. Results show the average expression levels from 3 independent experiments normalized to the expression of GAPDH.

Neurons were infected in the presence of ACV and cultured a further 10d in its presence. ACV was then withdrawn, and the cultures maintained an additional 15d. As a positive control for neuronal infection, some wells of neurons were infected without ACV treatment. At the end of the incubation period RNA was extracted from the neurons and the expression of transcripts for ORF61, ORF31, ORF63, and VLT exons 3–4 were measured by qRT-PCR (Figure 5). 

Neurons that did not receive ACV treatment were productively infected by all three viruses and expressed all 4 of the transcripts assayed for. In contrast, neurons that were infected in the presence of ACV showed greatly reduced expression of lytic RNAs from ORF31 and ORF61 while the RNAs for latency associated ORF63 and VLT were expressed at relatively high levels. The primers for ORF63 identified the portion of the transcript known to be also expressed as a fusion with VLT [8]. This data indicates that the mutant viruses were able to establish experimental latency in a manner indistinguishable from the parental virus and other recombinant VZV used in our laboratory. 

### 3.5. VZV Mutated in VZVsncRNA10 or Loss of VZVsncRNA12/13 Display Impaired Reactivation from Latency

After determining that the mutant viruses established latency in our model, latently infected neurons were treated with stimuli that we previously found to elicit reactivation and the generation of infectious viruses [6]. Neurons latently infected with VZVsnc10MUT, VZVsnc12-13DEL and VZV23GFP were maintained for 15d after ACV withdrawal and did not show GFP fluorescence. These cultures were then treated with LY294002 (10 μM) and phorbol ester phorbol 12-myristate 13-acetate (PMA) (40 ng/mL) and were incubated at 33 °C for a further 5 days. Reactivation of the virus in live cultures was followed by observation of GFP fluorescence indicating the expression of ORF23. 

**Figure 6:**
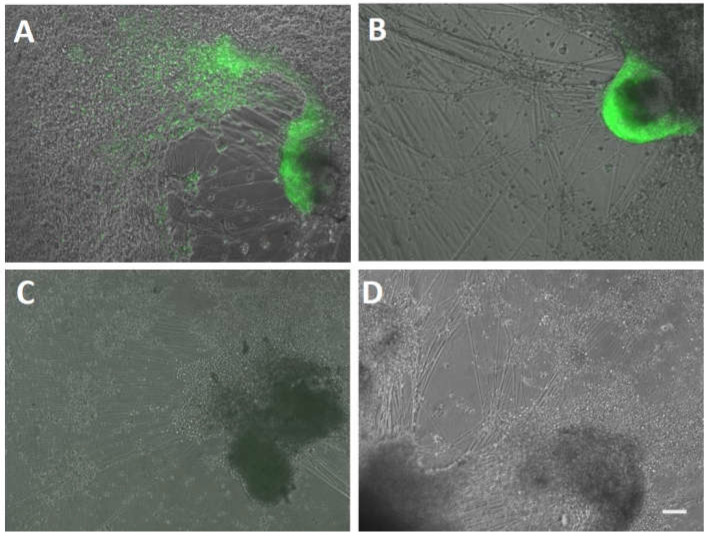
VZVsnc10MUT and VZVsnc12-13DEL are impaired for induced reactivation in hESC-derived neurons. Experimental latency was established in hESC-derived neurons using ACV treatment as described in [6] and the Methods. Ten to 15 days after ACV withdrawal there was no spontaneous reactivation as observed by lack of GFP fluorescence indicating production of the ORF23GFP fusion protein. The neurons then received a reactivating stimulus of P3K-inhibitor and PMA as detailed in the methods. Panels show a site of reactivated infection for VZV23GFP (**A**) which were detected in 3 wells of 9 infections performed in parallel to infection with the viruses mutant for VZVsncRNA. (**B**) The only well-containing neurons infected latently with VZVsnc10MUT that reactivated in three experiments including a total of 9 latently infected wells. No reactivation was observed in any of the 9 wells infected with the VZVsnc12-13DEL virus (**C**). (**D**) shows neurons in a mock-infected well receiving the reactivation stimulus. Three independent experiments for each virus VZV23GFP, MZVsnc10MUT, or VZVsnc12-13DEL were performed each including 3 wells infected with each virus. Size bar = 100 μm.

In three independent experiments, neurons infected with parental virus VZV23GFP expressed GFP in 1/3 of wells (3 wells GFP+ of 9 latently infected wells) of latently infected neurons (Figure 6A), consistent with results using a VZV expressing GFP fused to ORF66 [6]. In contrast, expression of GFP was only observed in one well in one of three experiments (that is, in 1 well GFP+ of 9 latently infected wells) with neurons latently infected with VZVsnc10MUT (Figure 6B). No GFP expression indicating reactivation was observed in 9 wells containing neurons latently infected with VZVsnc12-13DEL and given reactivation stimuli in the three experiments (Figure 6C). 

In order to confirm that the observed GFP expression from capsid protein ORF23 corresponded to complete reactivation including generation of the infective virus, we harvested neurons 5d after application of the chemical stimuli to the neurons and seeded them on naïve ARPE19 cells and monitored for the appearance of GFP-fluorescent foci of infection and cytopathic effect indicating a productive infection. Only re-seeded neurons that expressed GFP after receiving a reactivation stimulus were able to establish loci of infection, these eventually developed into plaques evident after crystal violet staining (not shown). Taken together, these results are consistent with the hypothesis that VZVsncRNA are involved in the reactivation of latent VZV.

## 4. Discussion

It has become clear that many biological processes are modulated by non-protein-coding RNA, particularly short RNAs. The short recognition sequence of these RNAs results in their ability to target multiple genes for regulation. miR is the most studied of these types of molecules, and it has been long known that viruses of the herpesvirus family encode many miR. The discovery that the HSV-1 LAT RNA encoded several miRNAs resulted in numerous studies to examine how they may regulate the expression of viral and host genes and the processes involved in lytic/latent decisions. miRNA have now been identified from multiple regions of HSV-1, and miRNA is encoded not only by LAT but also by transcripts of ICP0. Despite intense study, it is not yet known how these miR contribute to HSV-1 latency/reactivation [28]. Several miRNA loci have been deleted with minor or no effects, while mutation/deletion of other HSV-1 miRNA has influenced reactivation in rodent neurons infected with HSV-1 [29]. Most of the small non-coding RNA in VZV-infected cells and that encoded opposite to the VZV putative latency-associated transcript, VLT, are not predicted to fold into miR with current software tools. However, two sncRNA without classical miR structure have recently been reported to also be encoded by the HSV LAT, and these may affect the expression of the HSV receptor HVEM [30]. 

We have previously reported that chemically antagonizing these VZVsncRNA has significant effects on VZV replication and that combining several antagonists with them has stronger, additive effects. It was therefore somewhat surprising to not find significant effects on VZV replication in two cell lines infected with the VZV deletion or synonymous mutations viruses. It must be noted that the deletion of many VZV genes results in little to no effect on viral replication in cells in conventional monolayer cell culture, but when analyzed in the context of infection of human tissues in the SCID-Hu model, or in 3D in-vitro models such as intact skin these deletions have significant and dramatic effects on viral replication. Experiments are underway examining these and other VZV mutated in additional VZVsncRNA in such models. 

The location of VZVsncRNA10-13, along with the observation that HSV LAT-derived miR may play a role in latent infection/reactivation, stimulated our analyses into their possible roles in VZV latency and reactivation, using our hESC-derived neuron model. We reproducibly found that the two sncRNA mutant viruses were able to initiate infection and replicate productively in neurons and establish a latency-like state using ACV. When recombinant pOka-based virus expressing GFP is used for modeling latency, 1/3 of latently infected cultures (shown by in-situ hybridization for VZV DNA or VZV transcripts at long time periods after removal of virus/ACV) reactivate when the appropriate stimulus is provided [6]. This reactivation was productive, as evidenced by the generation of GFP-viral fusion protein and its spread over time to additional neurons, but also by the and the ability of the reactivated neurons to infect naïve ARPE19 cells. In the present study, we found that synonymous mutations in VZVsncRNA10 reduced the ability of VZV to reactivate in response to a stimulus, while deleting 12 nt common to VZVsncRNA12–13 eliminated reactivation altogether. Ideally, the specificity of these effects should be tested by restoring the VZVsncRNA by transfection of agonists. Unfortunately, transfection to neurons is not experimentally feasible. An alternative method of introduction of the VZVsncRNA sequences would be the development of AAV expressing them, and current experiments our laboratory are working out details of providing VZVsncRNA sequences with AAV serotypes that infect neurons (see [26] in this volume).

We still do not know the mechanism of action of the VZVsncRNA that do not seem to be miR, and additional studies will be necessary to elucidate how they exert their effects on reactivation and other aspects of VZV infection. We have observed that ORF61 and one of the VLT transcripts appear to be present at approximately the same levels in productively and latently infected neurons infected with the mutant VZV as in the parental virus (Figure 5). However, because the mutations introduced are in ORF61 and its 5′UTR, it is important to determine the effects of the mutations on ORF61 protein expression and localization as well as other VLT transcripts, and experiments addressing these issues now ongoing.

The current varicella-zoster vaccine given to children results in latent infection of peripheral neurons that can reactivate, albeit at a lower frequency than that of wild-type virus strains. Recent experiments in vitro [6] and the clinical experience show that vOka is impaired for reactivation, and usually results in milder disease when it occurs. However, our findings that deletion of 12 nt that are not required for viral replication and establishment of an immune response, but which can apparently abrogate reactivation, suggest the deletions generated could form the basis of a safer vaccine strain virus that is able to replicate in order to generate an immune response but unable to reactivate. The strategy of developing a VZV vaccine by making mutations modulating neuronal infection was recently published [31], utilizing a VZV mutant for ORF7, previously shown to impact VZV replication in skin and neurons [32]. This recent study demonstrates the interest and importance of developing safer vaccines for HZ and varicella disease.

## Figures and Tables

**Table 1:** Oligonucleotides used for making mutant viruses.

S.N.	Primer Name	Primer Sequence (5′→3′)
1	VZVsncRNA10MUTFW	CTTTACTCGGATGGCTTGATGATCAACTTGCGGACTGTACCAGCCTGAGATAGTCGAACCAACAAAAATGTTGATATAGGATGACGACGATAAGTAGC
2	VZVsncRNA10MUTREV	GCCCGTAAATACCTATATAGTTTAATATCAAACATTTTTGTTGGTTCGACTATCTCAGGCTGGTACAGTTCCGCAAGTCAGGGTAATGCCAGTGTTAC
3	SNC1213DelFW	GCTACCGCCCGCTAATATGGTATCCATGGTAACAACTGGCTGTATTCTACCAAACACGTAGCAGAACTGAGGATGACGACGATAAGTAGG
4	SNC1213delRev	TCACAATTTAGAACGCATGGCAGTTCTGCTACGTGTTTGGTAGAATACAGCCAGTTGTTACAGGGTAATGCCAGTGTTAC

**Table 2:** Primers used for detecting transcripts in VZV-infected cells by qRT-PCR.

ORF	FW Primer Sequence (5′→3′)	Rev Primer Sequence (5′→3′)
ORF31	CCGTGGGATTATTGGTTTTG	CGACGGTTCAGTGTTTTGTG
ORF61	AAAGCCTGACTTTTTGGGGT	CAAACCTGGACCTGGAAAGA
ORF63	ATTGAGGCGCCGAATGTTC	CTTCACCACCATCATCAGATACG
GAPDH	CACATGGCCTCCAAGGAGTAA	TGAGGGTCTCTCTCTTCCTCTTG
VLT exon 3–4	TGGACGATCACGGTAGTCCT	CGGAAAAACCATGCCGTGTT

## Data Availability

The data in this study are available in this article, as well as upon reasonable request from the corresponding author.
